# Time for global health policy and research leaders to prioritize endometriosis

**DOI:** 10.1038/s41467-023-43913-9

**Published:** 2023-12-04

**Authors:** Linda C. Giudice, Andrew W. Horne, Stacey A. Missmer

**Affiliations:** 1 Distinguished Professor, Center for Reproductive Sciences, Center for Reproductive Health, Department of Obstetrics, Gynecology and Reproductive Sciences, University of California, San Francisco, San Francisco, CA USA; 2 Professor of Gynaecology and Reproductive Sciences, EXPPECT Edinburgh and Centre for Reproductive Health, University of Edinburgh, Edinburgh, UK; 3 Professor of Obstetrics, Gynecology, and Reproductive Biology, Michigan State University, Grand Rapids, MI USA; 4 Adjunct Professor of Epidemiology, Harvard T.H. Chan School of Public Health, Boston, MA USA

**Keywords:** Public health, Reproductive disorders

## Abstract

Endometriosis is an incurable, under-diagnosed, systemic inflammatory disease affecting millions world-wide. Common symptoms include life-impacting pain, gastrointestinal/urinary symptoms, excessive fatigue, and infertility. Global public health policies are urgently needed to promote awareness, implement multidisciplinary care, and fund research for aetiology, biomarker discovery, and effective therapies for symptoms associated with endometriosis.

## Patient and public health impact of underinvestment in endometriosis

**Fig. 1:**
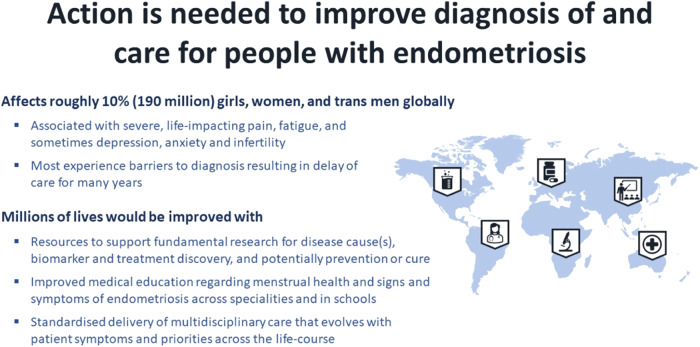
Endometriosis is a global disease needing global solutions. Basic research is key to understanding its causes and molecular and genomic underpinnings, leading to novel diagnostics and therapeutics to alleviate symptoms and ultimately discover a cure. Education of professional and lay communities and leaders throughout the world about endometriosis and its intersection with social determinants of health are key components to improve the lives of those affected. Moreover, multidisciplinary care is essential for holistic management of this complex and multi-dimensional disorder.

Defined by endometrium (uterine lining)-like tissue outside the uterus, endometriosis is a little known, common, hormone-dependent, inflammatory disorder currently diagnosed by surgical or radiologic visualisation of disease^1^. While mainly associated with life-impacting pelvic pain, painful menstruation and sexual intercourse, infertility, fatigue, and depression, those affected also have higher risk of non-reproductive sequelae, including high blood pressure, cardiovascular disease, autoimmune conditions, gastrointestinal and urologic symptoms, multifocal pain, migraines, and ovarian and breast cancer^1^ (Fig. 1). Endometriosis affects ~10% of persons with a uterus, commonly striking teens and persisting across the reproductive life span (and sometimes beyond), greatly impacting personal relationships, educational and employment opportunities, and quality of life^2,3^. Risk of developing disease is ~50% genetic, with no specific “endometriosis gene” or consistently identified environmental triggers^1^.

Reliance on surgery and marginal advances in medical therapies for symptom relief maintain delayed diagnosis and insufficient care for many^1^. Moreover, menstrual stigma, pain in women, sexism, and racism are impediments globally for life-improving care^3,4^. Lack of awareness and/or discomfort regarding menstrual health remains surprisingly prevalent among healthcare providers^3,4^.

Clinical specialties are commonly siloed, which diminishes recognizing and addressing endometriosis in clinical practices and specialist care outside gynecology^1,3^. Siloed structures also negatively impact awareness and applying cutting-edge technologies to multidisciplinary scientific discovery of causes, diagnostics, and treatments for endometriosis^3–5^. Impediments to determining aetiology and pathophysiology, classifying clinically-relevant subphenotypes, diagnosing, and treating endometriosis include socio-cultural factors that continue its obscurity among practitioners and the public^1,3,4^. Here we summarize challenges in endometriosis care and advancing research in this space.

A primary hypothesis for pelvic endometriosis establishment is through transplantation of endometrial tissue refluxed through the oviducts during menses, attaching to and invading pelvic organs, and eliciting a profound inflammatory response and fibrosis^1^. This is replicated in experimental rodent models that utilize seeding with heterologous or homologous endometriotic tissue^7^. However, this paradigm is insufficient, as nearly all persons with uteri experience “retrograde menstruation” and rodents do not menstruate. Thus genetic, epigenetic, hormonal, immunologic factors, endometrial stem cells, and coelomic metaplasia, have been implicated in establishment/survival of lesions (with pathway-informative somatic mutations)—complementing the theory that retrograde menstruation plays a causal role^1,4^. While mechanisms underlying this complex pathophysiology are slowly being elucidated, a comprehensive understanding of disease origins is wanting. This has stalled comprehensive animal models with disease fidelity—hampering research on endometriosis overall^6^.

Mechanisms underlying endometriosis-associated pain are complex^7^. Key to establishing lesions and activating peripheral pain pathways are new blood vessels and nerve sprouting (neuroangiogenesis)^7^. Also, in the central nervous system, brain volume and regional biochemical alterations accompany chronic endometriosis-related pain^7^. Endometriosis is also associated with infertility—attributed to scarred Fallopian tubes, diminished egg quality, and/or disrupted hormone signaling within the uterus^1,3,4^. Improved understanding of endometriosis pathogenesis and its complex and heterogenous manifestations and physiologic impacts are essential to develop improved diagnostics and patient-centred therapies.

## Heterogeneous presentation of endometriosis impedes diagnosis and treatment

Most pelvic endometriosis comprises three subtypes: superficial peritoneal, ovarian cysts (“endometrioma”), and deep disease^1^. Symptom manifestation and severity vary, and underdiagnosis is common due to erroneous symptom normalization and overlap with other diseases^8^. Recently, refined imaging techniques identify, with high accuracy, ovarian and deep disease, but reliance on surgical visualisation for the most common superficial peritoneal lesions continues to impede timely diagnosis^3,4^. Surgery or imaging requires access to healthcare services, which varies significantly across socio-economic and racial/ethnically underserved groups and geographies^4,9^. Average diagnostic delay is 7 years after symptom onset, with delays >10 years not uncommon^4,9^. There remains no reliable non-invasive biomarker of any endometriosis subtype^4^.

Treatment of pain symptoms falls broadly into surgical removal of disease and associated adhesions and hormonal suppressive therapies, including combined oral contraceptive steroids, progestins, gonadotropin releasing hormone analogues, androgens and aromatase inhibitors^10^. Medical therapies that decrease oestrogen (or counter oestrogen action) are prescribed, because steroids play a key role in the pathophysiology of endometriosis^1^. Unfortunately, both approaches are suboptimal. Surgery is associated with recurrence rates up to 50% within 5 years, and contraceptive hormone treatments often have unacceptable side effects and are counterproductive to fertility goals^10^. Geographic and financial barriers to accessing treatment from endometriosis-trained healthcare providers are common^9^. Surveys of patients consistently highlight symptom relief and improved medical therapies that do not limit fertility as a top priority for research^3^. Historically, pharmaceutical companies were reluctant to develop new drugs for endometriosis, and focus has been on variations of hormonal, anti-inflammatory, or repurposed therapies^11^, lacking revolutionary impact. While novel treatment discovery is essential, clinical trials for endometriosis have been plagued by widespread variations in outcome reporting, and only a fraction of completed trials is published^11^.

Emerging chronic pelvic pain-focused therapies that include considerations of neuropathic and nociceptive pathways have not been adequately studied^12^. Those with persistent/recurrent pain have a high rate of hysterectomy^12^ which does not eliminate pain recurrence and may heighten risks for multiple conditions later in life^1,10^. Further, commonly reported life-impacting concerns including fatigue and impaired sexual functioning have yet to be targeted for treatment among patients with endometriosis. A critical impediment to discovery of novel diagnostics and treatments and personalized approaches is the lack of a prognostically correlated classification of the highly heterogenous presentation of endometriosis lesion types and symptoms, which includes variation and evolution within patients across the life-course^3,4^.

## Endometriosis funding landscape and the socioeconomic cost of underinvestment

The first economic study (2011) revealed the average cost of endometriosis was ~€9579/woman/year (€6298 lost work-productivity, €3113 direct health care costs)—similar to diabetes, Crohn’s disease, and rheumatoid arthritis^13^. However, unlike these well-known diseases with similar socioeconomic impact and burden but considerably lower prevalence in the general population, there is relatively little investment in research into causes and disease mechanisms of endometriosis^5^. Recent governmental attention has included an increase in NIH funding for endometriosis research to $16 M (0.04% of the total NIH budget) for the year 2022 ($2/person with endometriosis/year), while Crohn’s disease received $90 M ($130/person with Crohn’s/year)^14^. Data consistently demonstrate that female-specific conditions are disproportionately underfunded^5^. Further, female reproductive conditions are largely absent from open access reference databases on which much of advanced biomolecular data science relies (e.g., ENCODE, NIH Roadmap epigenomics, GTEx, TissueNexus), impeding novel discovery, although recent endometrium and endometriosis single-cell transcriptomic data should soon appear in the Human Cell Atlas^4^.

## Commitments to endometriosis research and care and moving forward

Despite identification of endometriosis as a chronic inflammatory pain condition with multi-systemic symptoms and co-morbidities^1,8^, as a disease associated with menstruation and pelvic pain, it is still generally considered a gynaecologic disorder. This reductive concept has resulted in lowered healthcare system and research prioritization, limited integration, and impeded translation of rapidly developing scientific, multi-omic, genetic, bioengineering, and clinical discoveries in pain and inflammation. Their application would hasten identifying shared and unique features of endometriosis, leading to novel therapeutics and multi-disciplinary approaches to symptom management.

Essential to improving diagnosis and care for endometriosis patients is ensuring they are heard and believed when expressing their symptoms to themselves, their personal support networks, and especially to healthcare providers^4,10^. The 2021 WHO Endometriosis Fact Sheet (https://www.who.int/news-room/fact-sheets/detail/endometriosis) is a welcome leap forward to increase awareness globally. We posit that education and improved awareness including menstrual wellbeing curricula within schools for students of all gender identities could overcome menstruation taboos, ensure understanding of a ‘normal’ period, and when to seek help for distressing symptoms. Improved medical education regarding menstruation and endometriosis signs and symptoms could be ensured with thoughtful targets, e.g., including questions about menstrual health and non-malignant gynaecologic conditions on medical training board examinations, and larger scale initiatives such as revision of didactic teaching and medical curricula to prioritize menstrual health as critical to patient care.

Once diagnosed, individuals with endometriosis require personalised, multimodal, interdisciplinary treatment across the life-course to meet challenges of the evolving disease and changing patient priorities. First proposed in 2006, this novel care approach is optimally delivered through specialist centres comprised of integrated services including gynaecologists, endometriosis specialist nurses, and experts in imaging, pain medicine, psychology, physiotherapy, fertility, colorectal surgery, urology, and gastroenterology^15^. Whilst specialist centres have been successfully implemented in the UK and a few European countries (e.g., Denmark, Germany, France), this is not standard of care worldwide. Importantly, care delivery models incorporating mobile and digital technologies, and including primary care providers, specialists, pharmacy-based, community-based, and risk-prevention approaches, offer great opportunities and need development and investment^4^. For example, the Australian National Action Plan for Endometriosis launched in 2018 with $58 M additional funding in 2022 supports a 4-year plan to fund research and establish specialist endometriosis and pelvic pain clinics in every state and territory. Restructuring similar models globally could fulfil personalized care in all regions.

As delayed or undiagnosed endometriosis leads to compromised health, promoting awareness, improving access to care, and implementing multidisciplinary care paradigms are urgently needed in global public health policies. Equally urgent and important are prioritizing and committing resources to support fundamental research and biomarker discovery to shorten the protracted time to diagnosis and provide effective, long-term therapies for this chronic and debilitating disorder to the benefit of millions world-wide.
